# Medical Journals and Medical Criticism

**Published:** 1851-10

**Authors:** W. S. Chipley

**Affiliations:** Lexington, Ky.


### WESTERN JOURNAL

OF

### MEDICINE AND SURGERY.

OCTOBER, 1851.

ART. I—*Medical Journals and Medical Criticism.* By W. S. CHIPLEY, M. D., of Lexington, Ky.

Periodical medicine has exerted a wonderful influence for the space of more than a century. If it cannot command our veneration by its antiquity, it must be confessed to be exceedingly interesting and instructive in its comparatively brief history. It has been productive of much good and has, not unfrequently, wrought great wrong.—It has often puffed into magnitude the merest pretender; while, for a time, it has been but too successful in throwing obscuring shadows over the really meritorious—repressing their energies, and casting a damper upon the fires of their enthusiasm. It has scattered broad cast over the civilized world a multitude of valuable facts, the aggregation of which has developed important practical principles; but with the precious grain have been sown tares, in the form of "false facts" which, like *ignes fatui*, have led many a luckless wight from truth far into the dominion of error. It has been made the instrument for the overthrow of prevailing doctrines, and for the propagation of many of the wildest fancies that the perverted intellect of man ever conceived. At one time the vehicle of visionary conceits, that dissolved on the first touch of reason and philosophy, leaving not a wreck behind; at another it has been the medium through which have been developed some of the noblest conceptions of human genius, the most valuable improvements of the human intellect, and the most useful practical principles ever derived from human experience.

Its history is indeed rich in interesting materials for a graphic pen, and if fully and fairly traced would be one of the most acceptable and instructive pictures that could be presented to the profession. For our present purpose, a mere allusion to its early dawn in our mother country, and its growth in this, will be sufficient.

The first medical periodical in Great Britain, "*The Medical Essays and Observations, by a Society in Edinburg*," was issued in 1730. Similar enterprises were soon undertaken at different points; some destined to languish through a brief and feeble existence; others, to live long, and to add much to the sum of accumulating knowledge, and lustre to the reputation of their illustrious conductors. In the course of one hundred years so considerable was the increase of the number of periodicals devoted to the interests of the healing art that a distinguished journalist declared, "that a time, it is possible, may come, when a medical journal will issue with each clock that strikes, from nine in the morning till ten at night." Nor would this consummation be anything but desirable, if the quality kept pace with the quantity. For this mode of instruction has ever been since its origin deservedly popular with the profession; the information conveyed to it by this means is so fresh, and in a form so attractive, that not a few practitioners peruse medical periodicals with avidity, who are almost utter strangers to systematic works.

When conducted or supplied with material by such men as Monro, Douglas, Barry, John Armstrong, Pringle, Fothergill, Duncan, James Johnson, or by such as Mitchell, Miller, Smith, Coxe, Hosack, Francis, Caldwell, Chapman, and a host of others, in our own country, it would be difficult to over-estimate their importance or to exaggerate their value. But when they sink down, and become the mere organs of some medical school, on which they are designed to confer unmerited consequence, or are issued for notoriety by individuals, who seek self-aggrandizement by unblushing self-laudation, they are perverted from their legitimate and valuable purposes, by their incompetent, and not unfrequently, dishonest conductors, who would indiscriminately strike truth to the earth if interposed between them and their object.

The reason given in 1730, for the presentation of a medical periodical to the profession in Great Britain, has been reiterated by the projectors of almost all of its numerous successors in every part of the cultivated world. The editors say: "No complaint is more general among those who apply to the study of any liberal science, than their being under a necessity of perusing such numbers of publications as are wrote, or the several parts of each of them—a labor that can have no end, since one book serves only as an introduction to another, while a few pages might contain all that is new or valuable in *most of them*."

This is certainly a good and sufficient reason for the establishment and continuation of periodical medicine, especially, if it be true, as was charged many years ago, that the greater number of medical publications are written "for anything rather than with a view to promote the science of medicine."

With a brief notice of periodical medicine in this country, we may perceive to what extent our journals have subserved the purpose of giving to the profession all that our books contain that is new and valuable; and how far they are to be relied upon, in their criticisms of new works, in directing the practitioner in the purchase of valuable and reliable ones, and in protecting him from imposition by mere pretenders in authorship.

The first medical periodical issued in America, the "Medical Repository," made its appearance in New York in 1797, and was edited by Drs. Mitchell, Miller and Smith. This journal was conducted with signal ability, and is, to this day, a valuable work of reference. Stored with innumerable facts, well observed and faithfully recorded, and enriched with the thoughts of some of the first intellects that have adorned our profession, it can never cease to be a source of just pride to those who look back to that early period of our national history. Little more than half a score of years had elapsed since the termination of that eventful struggle which had severed our connection with a country, to which we had naturally looked for instruction in every department of science and literature; yet at that early period, we have evidence of a vigor of intellect and independence of thought that would be highly creditable to any people.

In 1804, the "Philadelphia Medical Museum" was presented to the profession, under the auspices of John Redman Coxe, M. D., who deemed it proper to apologize for undertaking to conduct such a work, when so good a journal as the "Repository" already existed. But no one can consult the pages of this periodical, without a feeling of gratitude to Dr. Coxe, for the immense amount of valuable matter which he has stored up in the volumes of his journal, and much of which will be held in high esteem as long as medicine is cultivated as a science.

"The American Medical and Philosophical Register" made its appearance in 1810, conducted by those distinguished cultivators of medical science, Drs. Hosack and Francis.

In 1812, Boston followed her sister cities into this interesting field, giving to the profession "The New England Journal of Medicine and Surgery."

Up to this period, and for some years after, the medical journals in this country were few in number, but they were conducted by men of eminent ability and enlarged experience, aided by contributors who would adorn any age. With an eye single to the advancement of medical science, the improvement of their professional brethren, and the welfare of society—with truth only for their guide, and uninfluenced by devotion to this or that clique, or the interest of this or that school, they bent all the energy of their vast intellects to the study of nature, and to the discovery of those broad principles which are to direct the medical philosopher in the great struggle against disease and death. Having no favorite but truth—no system but nature, their works will endure while truth is admired and nature is consistent.

In the department of criticism, there was a manifestation of a candor, truthfulness, an elevation of thought, and a moral dignity, that inspired confidence, and may have furnished the original of the poet's picture when,

> "Critic was a glorious name,
> Whose sanction handed merit up to fame;
> Beauties as well as faults he brought to view—
> His judgment great, and great his candor too."

These great works have been succeeded by a host of journals, professing the same objects; they are springing up in every section of our wide extended country; every sect, school and party in medicine, and even individuals deem it a matter of importance to have a monthly or quarterly to sustain their peculiar interests and views. So numerous have they become, that the prediction of the English journalist is now in a fair way of being verified in this country; and we cannot but regret that while many of them are creditable to the literature of our native land, characterised alike by the scientific attainments of their editors and contributors, and the spirit of fairness, justice and impartiality that pervades their pages; there are others marked by a carelessness, indolence or incompetence that cannot fail to lower the literary reputation of the mass of the profession. There are still others that bear the stamp of a more discreditable spirit—that spirit that feels alone the impulse of envy and jealousy, or that seeks notoriety only as a means of mercenary gain. The advantages of criticism are admitted to be manifold, when governed by the pure principles of justice, and directed by an unwavering regard to the general good. But the critic to fulfil his function with honor to himself and advantage to his reader, must possess many of the loftiest and most ennobling qualities of our nature. In addition to varied information, he must possess a penetration that can pierce the depths of the subject he undertakes to examine, a clear conception of what is lofty and sublime; what low, mean, and trivial, what appropriate and useful, or improper and hurtful. Discarding the baser qualities of our nature—envy, jealousy and hatred, he must bring to the consideration of his subject a delicate and discerning taste, chastened by that charity that delights to dwell upon truth, merit, and beauty, rather than to seize upon the errors, blemishes and faults that, after all, may be but specks that mar the perfection of a work, and prove it to be the production of man.

The critic who is suitably impressed with the importance, responsibility and dignity of his high vocation, loves to cherish the truthful and good, and encounters the antipodes of these only with pain and regret. While, as in duty bound, he is sometimes required to censure the work, charity interposes between him and his author. If there be any good, though mingled with a mass of error, it is his duty to acknowledge the former, as it is his prerogative to condemn the latter.

He should never forget that he himself may be deficient in attention or penetration, or ignorant of some collateral branch of science, and be thus led to attribute faults to his author which belong alone to himself. He is wholly unfitted for the office of critic who does not partake of that enlarged and liberal spirit which actuated Socrates in his reply to one who asked his opinion of a work that he had just perused; "what I understand I find to be excellent, and therefore, believe that to be of equal value which I cannot understand."

There are some who seem to think that no criticism will be relished unless it be highly seasoned, not by candid and philosophical exposure of error, but by assaults of ridicule or denunciation of the author. This is a great mistake. The mass of the profession, who are actively engaged in the discharge of their arduous duties, and who have a conscientious regard to the welfare of those committed to their charge, look upon such exhibitions of temper and feeling only with loathing and disgust. Whenever criticism, especially of medical works, passes beyond the bounds of fair and generous scrutiny, and descends from its high position to deal in invective instead of facts—in ridicule instead of argument, the great mass of medical men will believe with an eminent writer, that "it is too often founded in self-conceit, prejudice and unworthy feeling."

While it is a duty to repel the intrusive impertinence of ignorant literary coxcombs, and to expose the empirical pretensions of the arrogant knave, the critic should never forget that his chief duty is to instruct, not to censure; least of all should he seek to feed his own vanity by striking at unresisting feebleness—to gratify his envy by seeking to drag others to his own level, or his malice by wantonly wounding the sensibility of his author. All such feelings and motives will be discarded by those who have a proper regard to the principles of justice, to their own honor and to the good of their readers. Gentle admonition of error may be beneficial to the author who elicits it, but the ill-natured ravings of a self-constituted and conceited critic can be productive of no good, and merits only the contempt of the virtuous and enlightened.

It has been truly said, that the critic who has little to hazard, is apt to strike with fearless impetuosity, and his blows are never more eager or wilder than when his head whirls with gazing far above him at the philosophic, quiet renown and immortality of his author.

There are numerous classes of critics. Some open a book only to look for pleasing flowers and enchanting prospects; charmed with these, they close their eyes upon any errors or faults that may fall in their way. This may be pardoned as an amiable weakness. But there is another class who open a book only to seek and dwell upon its faults—lost to every feeling of humanity and justice, they revel with delight on that which should give only pain—vitiated in taste, malicious in feeling, blind to the most striking beauties, and deaf to every sound of truth, their ravings are proportioned to the excellency, and their happiness may be measured by the defects of their author:

"Hot, envious, noisy, proud, the scribbling fry,
Burn, hiss, and bounce, waste paper, ink and die."

There is another class somewhat more discriminating. Their criticism takes its cast and complexion from the name that may happen to grace the title page.

If the author is a favorite, especially if he be an advocate of the critic's peculiar notions, he may calculate with certainty, on being besprinkled with praise without judgment, and adulation without measure. No assumptions of fact—no errors in argument—no obscurity in style or impropriety of diction will make any impression; but all meet the unqualified approbation of the single-minded critic.

But if the author has been so unfortunate as to have offended the critic, or entertains views dissimilar to his own, the picture is reversed; the facts are false or inapplicable—the deductions illegitimate—the style feeble and obscure—the diction low, mean and incorrect.

The sycophancy and injustice displayed by these gross and venal writers would be incredible to one who has not observed them. They dwell with an earnestness of which truth only is worthy, and a vociferation which betrays their falsity, on the faults of an individual whom they choose to regard as an enemy; and assiduously labor to conceal, and not unfrequently they warmly defend, the same offences in an idol or partisan. Both departures from a right line are detestable; yet they are but too often manifest in periodicals that ought to be devoted only to truth and justice.

It is lamentable to observe the complete mental prostitution of certain partisan writers, ardently engaged in palliating error, disguising truth, mimicing conviction, and misleading the unwary. The grossest adulation of a favorite, or of one from whom something may be expected, often flows from the same pen that traces the vilest slanders of those whose superior attainments arouse the rancorous feelings of little minds, who, knowing that they cannot rival, seek to destroy;—who, conscious that they cannot fairly win position, strive to attain it by flattery on the one hand and defamation on the other. There is, however, one consolation for able writers, happily expressed by the poet:

> "Good authors damn'd, have their revenge in this,
> To see what wretches gain the praise they miss."

I have known instances of persons of some fancy, but of little knowledge and less judgment, assuming the responsible duties of an editor of a medical journal, apparently for no other purpose than that of waging war against every thing and every body. Literary Corsairs, they make piratical depredations upon other periodicals, for every thing valuable that their own contains, while their editorial efforts are mainly limited to vituperative assaults on those who have the independence to think for themselves, and the manliness to speak their opinions without fear or favor;—or to personal detraction of those whose virtues, attainments and renown they may never hope to emulate. A little insight into the history of these unscrupulous Ishmaelites, will usually bring the conviction that they are actuated by feelings embittered by disappointed ambition, and that their envenomed shafts fly thick and fast in proportion as they have hoped without reason, and aspired without merit. They readily bring themselves to believe that the world has thought enough of them to stretch forth its mighty hand to crush their hopes, and to defeat their cherished aspirations, when the very reckless effort to avenge their fancied wrongs proves at the same time, the cause of failure to have been their own innate feebleness, and vindicate the justness of the decree that drives them to an obscurity from which they can escape only by becoming

"Fix'd statues on the pedestal of scorn."

I regret to say, that the multiplication of medical schools has given birth to another class of critics, who can see nothing good in any production emanating from any school to which they are not attached—and nothing but perfection in the bantlings of those with whom they are associated or hope to become connected. The rivalship of medical schools has thus been a fruitful source of evil, and has nurtured a bad spirit in the highest walks of the profession—has induced the most extravagant laudation of mere pigmies, and the defamation of the ablest teachers and writers that adorn the present age. There are some, alas too many, in the medical profession, who are too impatient to bide their time;—greedy of present self-aggrandisement, they do not hesitate to sacrifice future reputation to an ambition out of all proportion to their merit and acquirements.

Many of the medical critics of the present day are so manifestly tinctured with false taste, prejudice, envy and jealousy, as to remind one of the beautiful allegory of Dr. Johnson, in which Criticism is represented as the daughter of Labor and Truth. At birth she was committed to the care of Justice, and brought up in the palace of Wisdom. When about to visit our sphere, her preceptor and guardian conferred upon her a sceptre, which she bore aloft in her right hand; one end was tinctured with ambrosia and enwreathed with golden foliage of amaranths and bays; the other end was encircled with cypress and poppies, and dipped in the waters of oblivion. In her left hand was an inextinguishable torch, manufactured by Labor and lighted by Truth. A single gleam of this torch penetrated the dark labyrinths of sophistry, and pierced the veil which rhetoric too often throws over the deformities of error. A touch of one end of her scepter conferred immortality—of the other, oblivion. But the intermixture of good and bad, characteristic of all human productions, often rendered doubtful the question whether the work merited the one or the other. Criticism observed the conduct of Time, and was so well pleased with his decisions that she withdrew from the earth, with her patroness Astrea, and left Prejudice and False-Taste to ravage at large, as the associates of Fraud and Mischief; contenting herself henceforth to shed her influence from afar upon some select minds, fitted for its reception by learning and by virtue. Before her departure she broke her scepter; a part of the ambrosia was caught up by Flattery, and with equal avidity the lethean portion was seized by Malevolence. Flattery neither had nor desired light, but touched indiscriminately whatever Power and Interest happened to exhibit; the Furies furnished Malevolence a torch whose infernal lustre shed light only on faults.

Not a few critics of the present day seem to have possessed themselves of fragments from both ends of the broken scepter, and thus armed seek by flattery and malevolence to confer immortality, or to condemn to oblivion. But the scepter has lost its power, and Time passes his sentence at leisure, without any regard to their determination.

Incapable of producing any work of learning or genius, by which they may win a substantial reputation, these writers hang about the portals of fame, ever ready to raise the hue and cry, in which they are eagerly joined by Ignorance and Envy, in pursuit of their noble prey; or, conscious of their own weakness, and dependent upon the favor of those in power, they frown and flatter, and thus sometimes crawl to the goal of their hopes; their whole course, dictated by self-interest, is marked by injustice and insincerity. Temporary success may thus reward their unhallowed exertions, but, in the language of a medical philosopher, "the public is a stern, inflexible, and finally, unerring tribunal, before which it is unnecessary to plead the cause of merit, and useless to varnish infirmity." Friends may flatter and enemies may defame, but the public at large do justice, because they are far removed from the sphere of personal feeling and influence.

Another source of evil, is to be found in that species of criticism, modestly called a "notice" of a new book. No argument can be necessary to convince any one of reflection that, whether the work be meritorious or the reverse, justice cannot be done to the author, or to the reader, without an analysis. These notices are often written by men every way competent to judge, and with honesty enough to be impartial; but pressed by other engagements they too often endorse the book, because it bears a name known to fame, or express an opinion after an examination of perhaps only the preface or a single chapter.

In these notices we are furnished with no elements on which to base a judgment—we have simply the opinion of the critic, and a fair presumption arises that this is not the result of a careful perusal of his author.

The result is that, not unfrequently, the members of the profession are induced to purchase an inferior work, and to neglect another of great value.

I have known the readers of one medical journal to be seeking to procure a new volume; while those of another periodical would speak of the same work with the utmost contempt, as utterly valueless and unworthy of perusal. It will be readily perceived how this may occur, when it is considered how widely the medical critics differ in their estimates of the same book. A few examples will illustrate this point more clearly. In giving them, I do not wish to be understood as charging the writers with any improper motive, or with a design to do injustice to authors. These differences may have arisen from an honest difference of opinion, but, probably, more frequently from a want of time for a thorough examination and analysis of the book.

The following examples are from recent American medical journals, but such discrepancies are not peculiar to our periodicals; they abound also in those of Europe, but I have not thought proper to note them.

A western journal thus notices a work not long since published:—

"We have no doubt that this treatise will prove highly useful to the young practitioners of the south. The author seems to be an acute observer, a patient and indefatigable student, and, what is by no means unimportant, clothes his ideas in a clear, agreeable, and concise style. To the class of practitioners for whom it is designed, we most earnestly commend Dr. McGown's work, and advise them to study its pages carefully and deliberately. The northern physician who emigrates south, will find this work of great service," &c.

The same work is reviewed by a southern journal, and the following is a quotation from it.

After censuring the culpable omission of many of the most common diseases of the south, the reviewer says, "we might dismiss the work, but lest by steam or some '*accident*,' it should be carried out of the State in which it was written, we feel it our duty, as a journalist, to allude to its deficiencies, if not to expose its errors, both on account of its style and the principles inculcated.

"Instead of advancing the views and unfolding the principles which govern our practice in this region (the south) the author has confined himself to some common-place observations on a class of diseases as rare, in this latitude, as hydrophobia, the dance of St. Vitus, or any other accidental complaint.

"This feeble attempt to shed light upon a subject of which the author seems to know really but little," &c.

It would be difficult to find more pointed contradictions than are contained in these two notices of the same work. Highly commended by one, as useful and instructive, it is as bitterly denounced by the other, as incorrect in style, deficient in matter, and defective in practice. I do not know who is right and who wrong; but the effect is obvious; a student and reader of the first may be induced to procure the book and labor to master its contents, while a similar reader of the other journal would never think of wasting time on so trifling a production. One or the other is greatly wronged; for the one loses the time occupied in studying an incorrect and feeble effort, to develope the character and treatment of an important class of diseases, —a work defective both in style and principles; or the other is deterred from procuring the work of an acute observer, and patient and indefatigable student, who clothes his ideas in a clear, agreeable and concise style.

An eastern journal thus notices a work on the practice of medicine:

"The author's descriptions of disease are, in general, loose, vague, imperfect and confused," &c.

Of the same work, a western journal remarks:

"Much of the author's limited space small as it is, is taken up by matter which would have appeared to more advantage in the funny column of a country newspaper, than in a sober treatise on practical medicine. Nor can it be considered a very reliable guide for the student of medicine," &c.

Of the same work, another western journal says:

"The work before us bears evidence of extensive experience, careful observation, and judicious treatment of disease, and we have no doubt the young practitioner will find in it much valuable advice. We sincerely hope it may meet with a ready sale."

One journal says, of an Essay on Intestinal Auscultation, by Charles Hooker, M. D.:

"We have read with great interest the remarks of Dr. Hooker, and duly appreciate his contributions to this new source of diagnosis."

Of the same work another journal says:

"We can but think a learned professor might find some better occupation than chasing, per chance, a cubic inch of *sulphuretted hydrogen gas* through a tube, measuring more than thirty feet in length."

'Review of Chemistry for students,' by John G. Murphy, M. D., is thus noticed by one critic:—

"The author has accomplished his objects in a satisfactory manner, and the student will no doubt find his advantage in embracing the opportunity offered of lessening labor and saving time," &c. The errors are "so few in number as not to materially affect the value of the work as a whole."

Another thus speaks of the same work:

"So far as we have been able to examine this work, it appears to be admirably adapted to facilitate the study of chemistry by students," &c.

How different is the estimate of a cotemporary journal, in which the following occurs:—

"We are sorry to say that the imperfect style, the many inaccuracies, the confused and obscure expressions of his work, are rather calculated to mislead and confuse, than to enlighten and inform; and that the object of the author is totally defeated by his unfortunate attempt to make a digest of the productions and opinions of others in his own miserable style."

In a notice of an introductory lecture, one journal says:

"It is a spirited and vigorous production, graced with a fancy and animation of style, which we were not entirely prepared for, and we need hardly add, distinguished by an elevated tone on the subject of medical education."

Of this same 'lecture' a cotemporary critic remarks:

"The author presents a striking example of a mind so stored with heterogeneous knowledge, as to defy order, and trammelled by the very '*embarras de richesses.*' There is a provoking indefinitiveness running through the entire twenty-eight pages," &c. "In his usually obscure style," &c. "Several pages of solemn quiddities," &c. "Too much learnnig has not made him mad—but obfuscated the clear perceptions of mother wit," &c.

Examples similar to the above might be multiplied *usque ad nauseam*, if not *ad infinitum*; but enough have been given to exhibit the contrariety of opinion that exists amongst those to whom the whole profession look for guidance and instruction; and to demonstrate the uncertain sounds by which those, who are endeavoring to keep pace with the advance of medical science, are expected to be directed. The evil is manifestly enormous, and no one can contest the propriety of abating it. In common with the mass of the profession, I have felt its force, but abler and more influential men must supply the remedy. While the first minds in the profession, who have the commanding influence of talents and position, differ so widely in taste, principles, and practice, we cannot hope for the removal of that stigma so often cast in our teeth, that "doctors will disagree."

In the above remarks, I have carefully avoided making any application to any particular journal; and hope I shall not be considered as arrogating to myself the position of a censor. I aspire to no such office, but have sought to direct attention to what are very generally considered serious defects in many of the medical periodicals of the present day. Many of these periodicals are conducted by as honest, capable, and faithful men as can be found in any country—men of chaste and refined taste—of enlarged and liberal views—of varied and extensive acquirements, and of steady unwavering devotion to truth and justice. These, too, are aided by correspondents, possessing a tact for accurate observation and an ability to record their observations, that cannot fail to add to, and develope new resources against the numerous ills to which flesh is heir.

To these a liberal and generous support is due, and will rarely fail to be awarded. If they sometimes propagate error, no base motive stimulates to the act, and may be justly set down to the frailty of humanity. The good they do is immeasurable, and will live long after their unintentional errors are forgotten. Besides these, there are a few journals established and conducted by second rate, but ambitious and desperate adventurers, with whom the character and dignity of the profession are objects of secondary importance. For a time these inflict great and general injury; but, at no distant period, they will sink to their proper level, to be remembered only with merited contempt. Controlled by good sense, enlightened by careful study, actuated by a pure spirit of justice and devotion to our noble profession, the mass of our brethren, to their honor be it spoken, speedily discover the character and motives of journals of this character, and with commendable alacrity and unanimity, leave them to languish and expire in deserved neglect.

September, 1851.

